# Provision for Returned Sick or Wounded Soldiers

**Published:** 1862-05

**Authors:** 


### EDITORIAL DEPARTMENT.

### PROVISION FOR RETURNED SICK OR WOUNDED SOLDIERS.

The confident expectation of destructive battles taking place, in which the soldiers who have gone from Buffalo and Western New York, will be called to take part, and the great probability that in such event, greater facilities will be required for the proper care of the wounded, has called out a most lively interest in our citizens, that every provision be made in advance, which can possibly be required to render any who may be wounded, all the care and attention which can possibly be bestowed. A committee have been appointed to provide hospital supplies, nurses, clothing, &c., &c., and to make any other provision which may be necessary. This, we understand, is to be supplied to the regiments where they are, or may be stationed, and is certainly a very proper and praise-worthy undertaking; though we learn that Oliver G. Steele, Esq., Chairman of the Committee of Twenty, has received a despatch from Washington informing him that the efforts of our citizens for the relief of our sick and wounded soldiers must be directed through the Sanitary Commission, no independent action being entitled to official recognition. We have not yet heard of any provision being made for the *returned soldier;* disabled, mutilated, partially recovered, or otherwise helpless or incapacitated, who are eventually to be discharged, and return for care and treatment. This class will sooner or later make their appearance among us, and comprise cases of the highest importance and interest, not only to the philanthropist, the benevolent and the christian, but also to the physician and surgeon. The exposures of the camp and field, the influences of miasma and contagion will induce disease, which will not in many instances yield to the treatment of the military hospital, and will find its way back to our city and to the homes which generosity and benevolence may provide. The wounded will also receive their first dressings by the surgeons of the army, but secondary operations will be made at their homes, and provision for the treatment of the returned wounded soldier will constitute an important duty, and require early and liberal attention.

The surgery of this war will not be completed until years after peace has been restored; unextracted balls, diseased joints, ununited fractures, and the like, will be presented to our experienced surgeons at home, who will be intrusted with the care of these most important cases, from the choice of the patients or their friends.

If there should be a demand for volunteer surgeons, volunteer nurses, and extra hospital supplies, to be sent to the seat of action, there will soon be found necessity for homes, clothing, and supplies for those who will find their way to our city, and it would seem to us that one of the first and most important steps in providing for the probable wants of our soldiers would consist in preparing suitable and comfortable hospital accommodations, where they can be cared for, as the defenders of our country deserve. It may be possible that there is no occasion for anxiety about this matter, since Buffalo has ample hospital accommodations for several hundred should it be required; yet it may be well to consider this matter a little, for it is an important item in providing for the wants of soldiers. It will not perhaps demand so early and immediate attention, but it will require attention sufficiently soon and sufficiently long, and in the end will be found to constitute by far the greater necessity and obligation.

By this we do not mean that the soldiers are to be discharged because they are sick or disabled; certainly our Government will provide for them for the present; indeed is providing for them in most liberal manner. In this earlier and more pressing matter, Buffalo stands ready to provide in case they are allowed to do so for a large number of sick, wounded, or disabled men, and could fully accommodate them at the present Government prices. No more attractive accommodations could be desired than our City affords, and it will be only on account of inattention to these advantages if we are not charged with the care of many of those cases which can safely and properly be transported, to these healthy and comfortable homes.

---

The Medical Reform Bill has passed the lower House of Congress, and has since been subject to some amendments by a joint Committee of both Houses. As it now stands the changes which it produces are operative only during the present war.—*Med. Times.*

